# Reassessment Intervals for Transition From Low to High Fracture Risk Among Adults Older Than 50 Years

**DOI:** 10.1001/jamanetworkopen.2019.18954

**Published:** 2020-01-10

**Authors:** William D. Leslie, Suzanne N. Morin, Lisa M. Lix, Patrick Martineau, Mark Bryanton, Eugene V. McCloskey, Helena Johansson, Nicholas C. Harvey, John A. Kanis

**Affiliations:** 1 Department of Medicine, University of Manitoba, Winnipeg, Manitoba, Canada; 2 Division of General Internal Medicine, McGill University, Research Institute of the McGill University Health Centre, Montreal, Quebec, Canada; 3 Department of Community Health Sciences, University of Manitoba, Winnipeg, Manitoba, Canada; 4 Section of Nuclear Medicine, University of Manitoba, Winnipeg, Manitoba, Canada; 5 Harvard Medical School, Boston, Massachusetts; 6 Centre for Metabolic Bone Diseases, University of Sheffield Medical School, Sheffield, United Kingdom; 7 Mary McKillop Institute for Health Research, Australian Catholic University, Melbourne, Australia; 8 MRC Lifecourse Epidemiology Unit, University of Southampton, Southampton, United Kingdom; 9 NIHR Southampton Biomedical Research Centre, University of Southampton and University Hospital Southampton NHS Foundation Trust, Southampton, United Kingdom

## Abstract

**Importance:**

Fracture risk scores are used to identify individuals at high risk of major osteoporotic fracture or hip fracture for antiosteoporosis treatment. For those not meeting treatment thresholds at baseline, the optimal interval for reassessing fracture risk is uncertain.

**Objective:**

To examine reassessment intervals for transition from low to high fracture risk under guidelines-defined treatment thresholds.

**Design, Setting, and Participants:**

This retrospective cohort study included persons aged 50 years or older with fracture risk below treatment thresholds at baseline who had fracture risk reassessed at least 1 year later. Data were obtained from a population-based bone mineral density registry (baseline assessment during 1996-2015; reassessment to 2016) in the Province of Manitoba, Canada. Primary analysis was performed from May to June 2019. Analysis for the revision was performed in October 2019.

**Main Outcomes and Measures:**

The primary outcome was time to transition from low (below the treatment threshold) to high fracture risk (treatment-qualifying risk score using osteoporosis clinical practice guidelines strategies for Canada, the United States, and the United Kingdom).

**Results:**

The study population consisted of 10 564 individuals (94.1% women; mean [SD] age at baseline, 63.2 [8.2] years). At the time of reassessment (a mean [SD] interval of 5.2 [2.9] years between initial and subsequent fracture risk assessment), 690 (6.6%) had reached the fixed major osteoporotic fracture treatment threshold of 20%, 1546 (16.2%) had reached the fixed hip treatment threshold of 3%, and 932 (9.4%) had reached the age-dependent major osteoporotic fracture treatment threshold. Among those below 25% of the treatment threshold at baseline for each guideline, few (0%-3.0%) reached guidelines-defined high fracture risk at follow-up. In contrast, among those at the upper end of the scale for each guideline (75%-99% of the treatment threshold at baseline), 30.6% to 74.4% reached guidelines-defined high fracture risk. An increased number of clinical risk factors was associated with increased likelihood of reaching guidelines-defined high fracture risk (range for 3 guidelines, 17.1%-28.2%) compared with unchanged or decreased clinical risk factors (range for 3 guidelines, 3.3%-12.8%) (*P* < .001). Estimated time for 10% of the population to reach treatment-qualifying high fracture risk ranged from fewer than 3 years to more than 15 years.

**Conclusions and Relevance:**

The findings suggest that baseline fracture risk (as a fraction of the treatment threshold) and change in clinical risk factors can identify individuals with low and high probability of guidelines-defined high fracture risk during follow-up, thereby potentially helping to inform the reassessment interval.

## Key Points

**Question:**

What is the optimal reassessment interval to detect high fracture risk for those who do not meet the treatment threshold at baseline?

**Findings:**

In this cohort study of 10 564 individuals, after a mean interval of 5.2 years between initial and subsequent fracture risk assessment, a range of 6.6% to 16.2% of the population reached high fracture risk according to 3 guidelines-defined treatment thresholds. Simple criteria, such as baseline fracture risk as a fraction of the treatment threshold and change in number of clinical risk factors, were associated with transition to high fracture risk.

**Meaning:**

The findings suggest that baseline fracture risk and change in clinical risk factors can identify individuals with low and high probability of achieving a guidelines-defined treatment threshold and potentially help optimize the reassessment interval in routine clinical practice.

## Teaser

This cohort study examines reassessment intervals for transition from low to high fracture risk using a population-based bone mineral density registry from Manitoba, Canada.

## Introduction

Osteoporosis is characterized by susceptibility to fracture, with substantial health consequences for the individual and society.^1^ Bone mineral density (BMD) is associated with fracture risk but has low sensitivity, with most fractures occurring above the threshold for osteoporosis diagnosis (≥2.5 SDs below peak bone mass; T-score, –2.5 or lower).^2,3,4^ Fracture risk prediction algorithms that incorporate clinical risk factors independent of BMD have been developed to target high-risk individuals for treatment. At present, the fracture risk assessment (FRAX) tool is the most widely used and has been incorporated into more than 100 clinical practice guidelines.^5^ The FRAX tool evaluates 10-year risk of major osteoporotic fracture (MOF), defined as a composite of hip, clinical spine, distal forearm, and proximal humerus, and 10-year risk of hip fracture based on age, sex, body mass index, 7 additional clinical risk factors, and (optionally) femoral neck BMD.^6^ When used with BMD, the FRAX tool provides a higher sensitivity than BMD alone.^7^

Repeated BMD testing is commonly performed, with a substantial proportion of women receiving repeated tests within 2 years, a practice questioned by the Choosing Wisely campaign,^8^ speaking to the need for guidance to help clinicians make higher-value decisions regarding repeated BMD measurement.^8,9,10^ Studies examining BMD loss and transition to osteoporosis have provided insights into BMD testing intervals according to the level of baseline BMD.^11^ Subsequent analyses have evaluated time to reach clinically relevant fracture risk for those who fall below the treatment threshold at baseline^12^ and the doubling time in fracture risk,^13^ although these have not adequately considered baseline fracture risk relative to the treatment threshold, which would be expected to affect treatment eligibility. Specifically, one would expect a shorter interval for those just below the treatment threshold and a longer interval for those well below the treatment threshold. Change in clinical risk factors would also be expected to affect time to treatment qualification, with a shorter interval for those with new clinical risk factors and a longer interval for those with a reduction in clinical risk factors.

The current analysis was undertaken to examine reassessment intervals for transition from low (below the treatment threshold) to treatment-qualifying high fracture risk in routine clinical practice. We hypothesized that level of baseline risk relative to the treatment threshold and change in clinical risk factors are associated with the time to reach a treatment-qualifying high-risk level. Because treatment guidelines differ among countries and this may be associated with results, we examined 3 different osteoporosis practice guideline strategies for pharmacologic treatment thresholds: (1) fixed MOF with 10-year risk of 20% or greater (major determinant in Canadian guidelines^14^), (2) fixed hip fracture with 10-year risk of 3% or greater (major determinant under US National Osteoporosis Foundation guidelines^15^), and (3) an age-dependent threshold that plateaus after age 70 years corresponding to the MOF with 10-year risk for a woman who has already sustained a fragility fracture (major determinant according to the UK National Osteoporosis Guideline Group^16^).

## Methods

### Study Population

We performed a provincial registry-based cohort study to examine change in fracture risk score and treatment threshold qualification for individuals aged 50 years or older at the time of an initial fracture risk assessment including BMD (January 1, 1996, through March 31, 2015) and fracture risk reassessed 1 or more year later (extending to March 31, 2016). The study was approved by the Research Ethics Board of the University of Manitoba; data access was approved by the Health Information Privacy Committee of Manitoba Health; and the need for informed consent was waived in accordance with the Personal Health Information Act of Manitoba. The anonymized data extract used for this work was approved and created in 2018, and primary data analysis was completed from May to June 2019. Analysis for the revision was performed in October 2019. This study followed the Strengthening the Reporting of Observational Studies in Epidemiology (STROBE) reporting guideline.^17^

In Manitoba, Canada, dual energy x-ray absorptiometry (DXA)–based BMD testing is managed as an integrated clinical program.^18^ The program maintains a database of all DXA results, which can be linked with other provincial population-based computerized health databases through an anonymous personal identifier. The DXA database has completeness and accuracy in excess of 99%.^19^ Scans obtained before 1996 were excluded because of absence of at least 1 year of pharmacy data before entry. We excluded (1) nonresidents of the province, (2) individuals younger than 50 years at initial assessment, (3) those without femur neck BMD test data or other data required to calculate FRAX risk score at the initial assessment to subsequent reassessment, (4) those who received treatment (defined as a prescription of >3 months of oral or parenteral bisphosphonate, raloxifene, denosumab, calcitonin, teriparatide, or systemic estrogens), (5) those with a previous hip or spine fracture, and (6) those already qualifying as having reached a treatment-qualifying fracture risk threshold at the time of initial scan using the previous clinical practice guideline definitions.

### Bone Mineral Density Measurements and Fracture Risk

Hip DXA scans were performed and analyzed in accordance with manufacturer recommendations. Femoral neck T-scores (number of SDs above or below young adult mean BMD) were calculated from the third National Health and Nutrition Examination Survey (NHANES III; 1988-1994) white female reference values for fracture risk assessment following national and international guidelines.^14,20,21,22^ All reporting physicians and supervising technologists are required to maintain DXA certification with the International Society for Clinical Densitometry. The program’s quality assurance is under strict supervision by a medical physicist.^18^ The cross-calibrated instruments used for this study (1 DPX, 3 Prodigy, and 3 iDXA; GE/Lunar Healthcare) (between-scanner differences, <0.1 T-score) exhibited stable long-term performance (coefficient of variation, <0.5%). Short-term reproducibility (coefficient of variation) for femoral neck BMD from the multiple technologists was 2.3% (>400 repeated hip DXA scans performed within 28 days).

The 10-year risk of MOF and hip fracture risk were calculated using the fracture risk assessment tool, Canadian version (FRAX Desktop Multi-Patient Entry, version 3.7; Osteoporosis Research Limited), which was calibrated using nationwide hip fracture and mortality data.^23,24^ Predictions agree closely with observed fracture risk in our population.^25,26^ In brief, age, body mass index, femoral neck BMD, and other data required for calculating fracture risk with the FRAX tool were assessed from on-site measurements (height and weight) and information collected directly from individuals through the intake questionnaire at the time of each DXA scan.^27^ Questionnaire information was supplemented with population-based health care data (hospital discharge abstracts, medical claims diagnoses, and provincewide retail pharmacy database) as recently described, thereby ensuring complete information for almost all individuals.^28^ All fracture risk scores included BMD because this more accurately assesses fracture risk than do clinical risk factors or BMD alone and because there is no significant cost or limitation to repeating fracture risk scores based on clinical risk factors alone.^7^ If clinical risk factors changed between the initial fracture risk assessment and subsequent fracture risk reassessment, this was incorporated in the fracture risk scores.

### Outcomes

The primary outcome was time to transition from below the treatment threshold to a treatment-qualifying high fracture risk score according to the 3 osteoporosis clinical practice guidelines’ strategies: fixed MOF threshold of 20%, fixed hip threshold of 3%, and age-dependent MOF threshold.^14,15,16^ Analyses were stratified according to how close (or far) the initial fracture risk measurement was from the treatment threshold. This was operationalized as the fraction of the treatment threshold at baseline (<25%, 25%-49%, 50%-74%, and 75%-99%).

### Statistical Analysis

Descriptive statistics for demographic and baseline characteristics are presented as mean (SD) for normally distributed continuous variables, median (interquartile range [IQR]) for continuous variables with a nonnormal distribution, or number (percentage) for categorical variables. There were no missing data for the analytic cohort. Parametric (*t* tests) and nonparametric (Mann-Whitney test, χ^2^ test) methods were used to compare population characteristics according to subsequent treatment threshold qualifications. The Cochran-Armitage test was used to test for linear trend in reaching high fracture risk according to baseline risk categories. We examined the absolute and relative change in MOF and hip fracture risk over time according to change in the number of FRAX clinical risk factors (decrease, no change, or increase). Loess curve smoothing was performed and curves interpolated to 0.1-year increments. Kaplan-Meier curves were used to construct the cumulative incidence of reaching high fracture risk according to fraction of treatment threshold at baseline (<25%, 25%-49%, 50%-74%, and 75%-99%), and groups were compared using the log-rank test. Cox proportional hazards regression models were used to estimate time in years (with 95% CIs) for 10% of the population to reach the treatment threshold according to fraction of the treatment threshold at baseline and change in number of FRAX clinical risk factors (minimum of 1 year and maximum of 15 years at 0.1-year increments). Model covariates included fraction of treatment threshold at baseline, change in the number of FRAX clinical risk factors, and the 2-way interaction term of these variables. The proportional hazards assumptions were tested and confirmed by examining Schoenfeld residuals. The choice of 10% for transition to the treatment threshold was selected based on similar previous analyses.^11,12^ Separate analyses were conducted for the 3 different treatment strategies described previously. In sensitivity analyses, time for 5%, 20%, or 50% of the population to reach the treatment threshold was modeled, and change in the number of FRAX clinical risk factors was substratified as an increase of 1 vs 2 or more. Statistical analyses were performed with Statistica, version 13.0 (StatSoft Inc) and curve smoothing with figure generation using Sigmaplot, version 13.0 (Systat Software Inc). A 2-sided *P* ≤ .05 indicated statistical significance.

## Results

**Figure 1.**
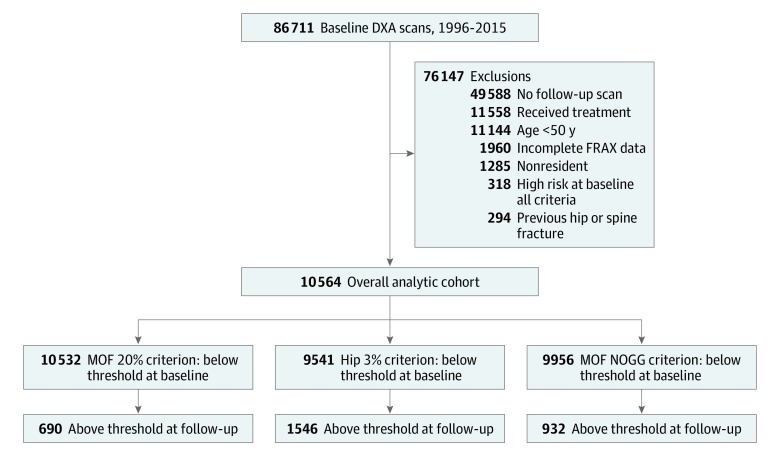
Flowchart of Individuals in the Cohort DXA indicates dual-energy x-ray absorptiometry; FRAX, fracture risk assessment; MOF, major osteoporotic fracture; and NOGG, National Osteoporosis Guideline Group.

The study population selection process is summarized in Figure 1. The analytic cohort consisted of 10 564 individuals contributing to 1 or more of the treatment paradigms: 10 532 fixed MOF, 9541 fixed hip, and 9956 age-dependent MOF. The mean (SD) age at baseline was 63.2 (8.2) years, and 94.1% were women. Median baseline MOF risk was 7.0% (IQR, 5.2%-9.8%), and median baseline hip fracture risk was 0.7% (IQR, 0.3%-1.5%).

The mean (SD) interval between initial fracture risk assessment and subsequent assessment was 5.2 (2.9) years. At the time of reassessment, 690 individuals (6.6%) had reached the fixed MOF treatment threshold of 20%, 1546 (16.2%) had reached the fixed hip treatment threshold of 3%, and 932 (9.4%) had reached the age-dependent MOF treatment threshold (Table 1). Median baseline fracture risk, as a fraction of the treatment threshold, was significantly greater among those who subsequently reached the treatment threshold and a designation of high fracture risk than among those who did not (fixed MOF treatment threshold: 0.69 [IQR, 0.52-0.85] vs 0.34 [IQR, 0.25-0.46]; fixed hip treatment threshold: 0.60 [IQR, 0.39-0.77] vs 0.15 [IQR, 0.07-0.30]; age-dependent MOF treatment threshold: 0.70 [IQR, 0.56-0.86] vs 0.49 [IQR, 0.43-0.60]; all *P* < .001). Over time, there was an increase in the total number of clinical risk factors, and this was significantly greater for those with transition to treatment-qualifying fracture risk than among those without (fixed MOF treatment threshold: 60.1% vs 20.4%; fixed hip treatment threshold: 40.4% vs 19.8%; age-dependent MOF treatment threshold: 69.5% vs 18.8%; all *P* < .001).

**Table 1.** Characteristics of the Study Population

Characteristic	Overall (N = 10 564)	Threshold at Follow-up								
		Reached Fixed MOF Threshold of 20%			Reached Fixed Hip Threshold of 3%			Above Age-Dependent MOF		
		No (n = 9842)	Yes (n = 690)	*P* Value	No (n = 7995)	Yes (n = 1546)	*P* Value	No (n = 9024)	Yes (n = 932)	*P* Value
Age, mean (SD), y	63.2 (8.2)	62.8 (8.0)	69.7 (8.2)	<.001	61.0 (7.1)	67.7 (6.8)	<.001	63.3 (8.0)	65.5 (10.0)	<.001
Women, No. (%)	9941 (94.1)	9232 (93.8)	677 (98.1)	<.001	7561 (94.6)	1466 (94.8)	.69	8466 (93.8)	902 (96.8)	<.001
Body mass index, mean (SD)^a^	27.4 (5.3)	27.4 (5.3)	26.8 (4.6)	.003	27.7 (5.4)	27.0 (4.8)	<.001	27.6 (5.3)	26.4 (4.8)	<.001
Previous fracture, No. (%)	783 (7.4)	600 (6.1)	159 (23.0)	<.001	435 (5.4)	185 (12.0)	<.001	420 (4.7)	129 (13.8)	<.001
Parental hip fracture, No. (%)	733 (6.9)	608 (6.2)	93 (13.5)	<.001	523 (6.5)	131 (8.5)	.006	318 (3.5)	91 (9.8)	<.001
Smoker, No. (%)	681 (6.4)	613 (6.2)	67 (9.7)	<.001	406 (5.1)	125 (8.1)	<.001	534 (5.9)	71 (7.6)	.04
Recent glucocorticoid use, No. (%)	506 (4.8)	464 (4.7)	38 (5.5)	.35	357 (4.5)	64 (4.1)	.57	353 (3.9)	48 (5.2)	.07
Rheumatoid arthritis, No. (%)	223 (2.1)	191 (1.9)	26 (3.8)	.001	147 (1.8)	45 (2.9)	.006	140 (1.6)	32 (3.4)	<.001
Secondary osteoporosis, No. (%)	1780 (16.8)	1694 (17.2)	83 (12.0)	<.001	1427 (17.8)	200 (12.9)	<.001	1544 (17.1)	133 (14.3)	.03
High alcohol use, No. (%)	25 (0.2)	S (<1.0)	S (<1.0)	.61	S (<1.0)	S (<1.0)	.34	S (<1.0)	S (<1.0)	.04
Femur neck T-score, mean (SD)	−1.2 (0.8)	−1.2 (0.8)	−1.9 (0.6)	<.001	−1.0 (0.8)	−1.7 (0.5)	<.001	−1.1 (0.8)	−1.8 (0.6)	<.001
Baseline 10-y MOF risk, median (IQR), %^b^	7.0 (5.2-9.8)	6.8 (5.1-9.2)	13.8 (10.3-17.0)	<.001	6.2 (4.7-7.9)	9.9 (8.2-11.7)	<.001	6.6 (5.0-8.9)	9.7 (6.7-14.7)	<.001
Baseline 10-y hip fracture risk, median (IQR), %	0.7 (0.3-1.5)	0.6 (0.3-1.3)	3.0 (1.5-4.6)	<.001	0.5 (0.2-0.9)	1.8 (1.2-2.3)	<.001	0.6 (0.2-1.3)	1.6 (0.6-3.7)	<.001
Interval, mean (SD), y^b^	5.2 (2.9)	5.1 (2.9)	6.3 (3.2)	<.001	5.0 (2.8)	6.4 (3.3)	<.001	5.1 (2.9)	5.9 (3.2)	<.001
Baseline MOF risk as fraction of fixed 20% threshold, median (IQR)^b^	0.35 (0.26-0.49)	0.34 (0.25-0.46)	0.69 (0.52-0.85)	<.001	0.31 (0.24-0.40)	0.49 (0.41-0.58)	<.001	0.33 (0.25-0.45)	0.49 (0.34-0.73)	<.001
Baseline hip fracture risk as fraction of fixed 3% threshold, median (IQR)^b^	0.19 (0.08-0.40)	0.18 (0.08-0.38)	0.51 (0.29-0.74)	<.001	0.15 (0.07-0.30)	0.60 (0.39-0.77)	<.001	0.17 (0.08-0.37)	0.29 (0.14-0.54)	<.001
Baseline MOF risk as fraction of age-dependent MOF threshold, median (IQR)^b^	0.50 (0.43-0.63)	0.50 (0.43-0.61)	0.75 (0.59-0.88)	<.001	0.48 (0.42-0.57)	0.57 (0.49-0.66)	<.001	0.49 (0.43-0.60)	0.70 (0.56-0.86)	<.001
Clinical risk factors, No. (%)										
Decrease	679 (6.4)	652 (6.6)	22 (3.2)	<.001	512 (6.4)	58 (3.8)	<.001	531 (5.9)	23 (2.5)	<.001
Increase	2430 (23.0)	2012 (20.4)	415 (60.1)	<.001	1585 (19.8)	624 (40.4)	<.001	1701 (18.8)	648 (69.5)	<.001

Abbreviations: IQR, interquartile range; MOF, major osteoporotic fracture; S, suppressed small numbers.

^a^ Calculated as weight in kilograms divided by height in meters squared.

^b^ Major osteoporotic fracture and hip fracture risk were computed using the fracture risk assessment tool with bone mineral density.

For those below 25% of the treatment threshold at baseline, few (range, 0%-3.0% for 3 guidelines) reached guidelines-defined high fracture risk at follow-up (eTable 1 in the Supplement). In contrast, for those at the upper end of the scale (75%-99% of the treatment threshold at baseline), 30.6% (age-dependent MOF threshold), 45.4% (fixed 10-year MOF threshold) and 74.4% (fixed 10-year hip fracture risk threshold) reached guidelines-defined high fracture risk. There was a statistically significant linear trend for overall change in clinical risk factors according to increasing fraction of treatment threshold at baseline (fixed MOF treatment threshold: 0.7% [<25% threshold], 2.5% [25%-49% threshold], 13.3% [50%-74% threshold], 45.4% [75%-99% threshold]; fixed hip treatment threshold: 3.0%, 18.7%, 47.8%, and 74.4%; age-dependent MOF treatment threshold: 0%, 10.9%, 30.6%, and 30.6%; all *P* < .001 for trend). An increase in the number of clinical risk factors was associated with increased likelihood of reaching guidelines-defined high fracture risk (range, 17.1%-28.2% for 3 guidelines) compared with a stable or decreased number of clinical risk factors (range, 3.3%-12.8% for 3 guidelines) (*P* < .001). Change in number of clinical risk factors was independently associated with the likelihood of reaching guidelines-defined high fracture risk at follow-up, with a statistically significant linear trend in all baseline risk categories except for those less than 25% of the age-dependent MOF treatment threshold at baseline because no one reached the treatment threshold.

**Figure 2.**
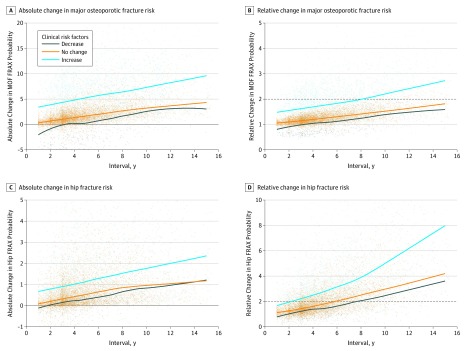
Major Osteoporotic Fracture (MOF) and Hip Fracture Risk Changes for Intervals Between Fracture Risk Assessments According to Change in the Number of Clinical Risk Factors Solid lines are Loess smoothed curves fitted to the dots, which indicate individual patient observations. Dashed line indicates a doubling in baseline fracture risk. FRAX indicates fracture risk assessment.

Figure 2 shows a gradual absolute and relative increase in fracture risk with increasing time interval, with a similar trend seen for individuals with a decrease, no change, or increase in clinical risk factors. The time to doubling in MOF risk was modified by change in the number of clinical risk factors. For individuals with a decrease or no change in the number of clinical risk factors, the time to high fracture risk exceeded 15 years and was 8.2 years when there was an increase in the number of clinical risk factors. For hip fracture risk, the doubling time was 7.7 years when there was a decrease in the number of clinical risk factors, 5.9 years when there was no change, and 2.3 years when there was an increase in the number of clinical risk factors.

**Figure 3.**
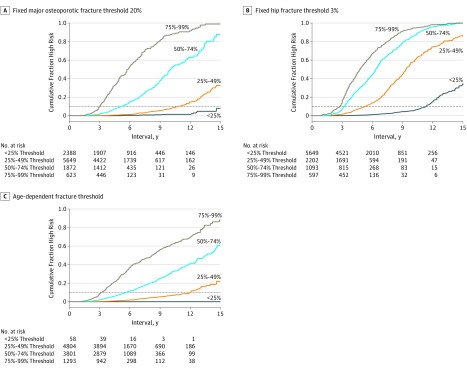
Cumulative Fraction of the Population Reaching High Fracture Risk According to Fraction of Treatment Threshold at Baseline Dashed line indicates point where 10% of the population reached high fracture risk.

The cumulative fraction of the population reaching high fracture risk was associated with baseline risk category for each of the 3 different treatment strategies, with shorter time to reach high fracture risk for those closer to the treatment threshold (Figure 3). The reassessment interval based on time for 10% of the population to reach guidelines-defined high fracture risk from the Cox proportional hazards regression models is summarized in Table 2. Greater baseline fracture risk and an increased number of clinical risk factors were associated with a shorter interval, whereas lower baseline fracture risk and a reduction in clinical risk factors were associated with an increased time interval to high fracture risk. For the fixed MOF treatment threshold, those with baseline risk less than 25% of the treatment threshold were unlikely to transition to guidelines-defined high fracture risk within the first 15 years. Even when there was an increase in the number of clinical risk factors, a cumulative fraction high risk of 13.3 years (95% CI, 11.9-14.5 years) was required for 10% of the population to reach high risk. Conversely, for individuals close to the treatment threshold (75%-99%), time for 10% of the population to reach high fracture risk was from 3 to 4 years even when there was a decrease in the number of clinical risk factors. Intermediate results were seen for the other scenarios. For fixed hip fracture treatment threshold, transition times were generally shorter, whereas for the age-dependent MOF treatment threshold, transition times were generally longer. Reassessment intervals for 5%, 20%, and 50% of the population to reach guidelines-defined high fracture risk are given in eTables 2-4 in the Supplement.

**Table 2.** Time for 10% of the Population to Reach High Fracture Risk According to Fraction of Treatment Threshold at Baseline and Change in Clinical Risk Factors

Change in Clinical Risk Factors	Baseline Threshold, Time to Threshold (95% CI), y^a^			
	<25%	25%-49%	50%-74%	75%-99%
**Fixed MOF Threshold of 20%**				
Decrease	>15 (>15 to >15)	>15 (14.5 to >15)	8.6 (6.7 to 13)	4.1 (3.6 to 5.5)
No change	>15 (>15 to >15)	>15 (>15 to >15)	7.1 (6.5 to 8.1)	2.9 (2.9 to 3.2)
Increase	13.3 (11.9 to 14.6)	7.1 (6.6 to 7.7)	3.5 (3.3 to 3.7)	2.9 (2.8 to 3.2)
Overall	>15 (14.6 to >15)	11.4 (10.3 to 12.3)	4.9 (4.7 to 5.4)	3.1 (2.9 to 3.3)
**Fixed Hip Fracture Threshold of 3%**				
Decrease	14.9 (13.1 to >15)	6.4 (5.3 to 8.4)	3.5 (3.2 to 4.3)	3.1 (2.9 to 3.7)
No change	14.9 (14.1 to 14.9)	5.5 (5.2 to 5.9)	3.2 (3.1 to 3.3)	2.7 (2.6 to 2.9)
Increase	8.1 (7.5 to 8.8)	3.6 (3.4 to 3.8)	3.1 (2.9 to 3.2)	3.0 (2.9 to 3.1)
Overall	11.2 (10.6 to 11.7)	4.6 (4.4 to 4.9)	3.2 (3.1 to 3.3)	2.8 (2.8 to 2.9)
**Age-dependent MOF Threshold**				
Decrease	>15 (>15 to >15)	>15 (>15 to >15)	10.4 (7.4 to 14.9)	5.2 (4.3 to 7.4)
No change	>15 (>15 to >15)	>15 (>15 to >15)	12.9 (11.8 to 14.7)	3.4 (3.2 to 3.6)
Increase	>15 (>15 to >15)	6.8 (6.1 to 7.7)	3.3 (3.2 to 3.5)	2.8 (2.6 to 2.9)
Overall	>15 (>15 to >15)	12.6 (11.8 to 13.6)	5.4 (5.1 to 5.7)	3.3 (3.2 to 3.5)

Abbreviation: MOF, major osteoporotic fracture.

^a^ More than 15 indicates that less than 10% of the population reached high fracture risk by 15 years.

Additional analyses showed that a greater increase in the number of clinical risk factors (≥2 vs 1) had an association with reaching guidelines-defined high fracture risk (eTable 5 in the Supplement). For those with an increase in the number of clinical risk factors of 2 or more, the time to doubling in MOF risk was 1.5 years, and for hip fracture risk, the time to doubling in MOF risk was less than 1 year (eFigure 1 in the Supplement). Age and sex, which were already considered in the baseline risk, made a negligible independent contribution in estimating reassessment intervals for transition to clinically relevant high fracture risk (eFigure 2 in the Supplement).

## Discussion

This analysis of a population-based clinical registry of individuals undergoing baseline and subsequent fracture risk assessment found that relatively few individuals (<20%) reached guidelines-defined high fracture risk after a mean (SD) of 5.2 (2.9) years (ranging from 6.6% for fixed MOF treatment threshold to 16.2% for fixed hip fracture threshold). Major variables associated with reaching treatment-qualifying fracture risk were the baseline level of risk (particularly when FRAX scores were 75%-99% of the treatment threshold) and an increase in the number of clinical risk factors. Together, these measures identified subgroups in which transition to guidelines-defined high fracture risk was unlikely for more than 15 years and others in which transition to high risk occurred before 3 years. Broadly similar patterns were seen for fracture risk strategies and thresholds that are included in the clinical practice guidelines for Canada, the United States, and the United Kingdom.^14,15,16^

Simulation analysis performed by Reid and Gamble^13^ found a doubling time in 10-year hip fracture risk of 5 to 6 years across a range of baseline assumptions, with a doubling time for 10-year MOF risk that exceeded 10 years. Gourlay et al^11^ analyzed the time for 10% of women who did not have osteoporosis at baseline to make the transition to osteoporosis. The estimated testing interval was 16.8 years for women with normal BMD and as short as 1.1 years for women with advanced osteopenia (BMD T-score, –2.00 to –2.49). A subsequent study extended these observations to examine the time for 10% of women to develop a treatment-level fracture risk score using US National Osteoporosis Foundation guidelines.^12^ Before age 65 years, postmenopausal women with a subthreshold fracture risk score at baseline rarely developed a treatment-level FRAX score. Time to a treatment-level score ranged from 7.6 years (ages 65-69 years) to 5.1 years (ages 75-79 years). Our study showed that estimated time to develop a treatment-level high fracture risk score was associated with baseline fracture risk and new clinical risk factors.

### Limitations

This study has limitations. Because the study population was derived from a clinical registry, the decision to reassess fracture risk was influenced by clinician and patient perception of an individual’s risk for fractures. However, this may have increased the relevance of our study to the clinical practice setting where such considerations are a standard part of patient care and decision-making because research cohorts may not reflect routine clinical practice.^29^ Our study did not consider competing mortality. We excluded individuals receiving pharmacologic treatment and those with previous major fracture (hip or clinical vertebral), which other researchers have addressed through a competing risk analysis^12^; the objective of this study was to examine the change in fracture risk in the absence of treatment (which would affect BMD loss) and those with previous hip or clinical vertebral fracture (generally recommended for treatment rather than further risk assessment). Our analysis of change in clinical risk factors did not consider their different weights in the FRAX tool because this can be directly modeled through the website. Also, the population was 98% white or of European ancestry, and the lack of racial/ethnic heterogeneity precluded a direct assessment of whether this modified our findings. Likewise, international FRAX models reflect underlying population differences in terms of fracture and mortality rates. The generalization of our findings to other populations and FRAX models is uncertain.

## Conclusions

The findings suggest that baseline fracture risk (as a fraction of the treatment threshold) and number of clinical risk factors can identify individuals with low and high probability of guidelines-defined high fracture risk during follow-up. This could potentially help to inform the reassessment interval.

## Notes

Supplement. **eTable 1.** Number (Percent) Reaching High Fracture Risk at Follow-up According to Fraction of Treatment Threshold at Baseline and Change in Number of Clinical Risk Factors (CRFs) **eTable 2.** Time in Years (95% CI) for 5% of the Population to Reach High Fracture Risk According to Fraction of Treatment Threshold at Baseline and Change in Number of Clinical Risk Factors (CRFs) **eTable 3.** Time in Years (95% CI) for 20% of the Population to Reach High Fracture Risk According to Fraction of Treatment Threshold at Baseline and Change in Number of Clinical Risk Factors (CRFs) **eTable 4.** Time in Years (95% CI) for 50% of the Population to Reach High Fracture Risk According to Fraction of Treatment Threshold at Baseline and Change in Number of Clinical Risk Factors (CRFs) **eTable 5.** Number (Percent) Reaching High Fracture Risk at Follow-up According to Fraction of Treatment Threshold at Baseline and Change in Number of Clinical Risk Factors (CRFs) Stratified as Decreases (–1), No Change (0), or Increase (+1, +2, or More) **eFigure 1.** Absolute and Relative Change in Major Osteoporotic Fracture (MOF) Risk and Hip Fracture Risk for Increasing Intervals Between Fracture Risk Assessments According to Change in the Number of Clinical Risk Factors (CRFs) Stratified as Decrease (–1), No Change (0), or Increase (+1, +2 or More) **eFigure 2.** Importance of Variables Predicting Transition to High Fracture Risk According to Fixed 20% Major Osteoporotic Fracture (MOF) Risk, Fixed 3% Hip Fracture Risk, and Age-dependent MOF Risk
