# Supplementary material for: Reassessment Intervals for Transition From Low to High Fracture Risk Among Adults Older Than 50 Years
Source: JAMA Netw Open. 2020 Jan 10;3(1):e1918954. doi: 10.1001/jamanetworkopen.2019.18954 (PMC6991318; doi:10.1001/jamanetworkopen.2019.18954)
Supplement: Supplement. — eTable 1. Number (Percent) Reaching High Fracture Risk at Follow-up According to Fraction of Treatment Threshold at Baseline and Change in Number of Clinical Risk Factors (CRFs) eTable 2. Time in Years (95% CI) for 5% of the Population to Reach High Fracture Risk According to Fraction of Treatment Threshold at Baseline and Change in Number of Clinical Risk Factors (CRFs) eTable 3. Time in Years (95% CI) for 20% of the Population to Reach High Fracture Risk According to Fraction of Treatment Threshold at Baseline and Change in Number of Clinical Risk Factors (CRFs) eTable 4. Time in Years (95% CI) for 50% of the Population to Reach High Fracture Risk According to Fraction of Treatment Threshold at Baseline and Change in Number of Clinical Risk Factors (CRFs) eTable 5. Number (Percent) Reaching High Fracture Risk at Follow-up According to Fraction of Treatment Threshold at Baseline and Change in Number of Clinical Risk Factors (CRFs) Stratified as Decreases (–1), No Change (0), or Increase (+1, +2, or More) eFigure 1. Absolute and Relative Change in Major Osteoporotic Fracture (MOF) Risk and Hip Fracture Risk for Increasing Intervals Between Fracture Risk Assessments According to Change in the Number of Clinical Risk Factors (CRFs) Stratified as Decrease (–1), No Change (0), or Increase (+1, +2 or More) eFigure 2. Importance of Variables Predicting Transition to High Fracture Risk According to Fixed 20% Major Osteoporotic Fracture (MOF) Risk, Fixed 3% Hip Fracture Risk, and Age-dependent MOF Risk [file jamanetwopen-3-e1918954-s001.pdf]

Supplementary Online Content

Leslie WD, Morin SN, Lix LM, et al. Reassessment intervals for transition from low to high fracture risk among adults older than 50 years. *JAMA Netw Open*. 2020;3(1):e1918954. doi:10.1001/jamanetworkopen.2019.18954

eTable 1. Number (Percent) Reaching High Fracture Risk at Follow-up According to Fraction of Treatment Threshold at Baseline and Change in Number of Clinical Risk Factors (CRFs)

eTable 2. Time in Years (95% CI) for 5% of the Population to Reach High Fracture Risk According to Fraction of Treatment Threshold at Baseline and Change in Number of Clinical Risk Factors (CRFs)

eTable 3. Time in Years (95% CI) for 20% of the Population to Reach High Fracture Risk According to Fraction of Treatment Threshold at Baseline and Change in Number of Clinical Risk Factors (CRFs)

eTable 4. Time in Years (95% CI) for 50% of the Population to Reach High Fracture Risk According to Fraction of Treatment Threshold at Baseline and Change in Number of Clinical Risk Factors (CRFs)

eTable 5. Number (Percent) Reaching High Fracture Risk at Follow-up According to Fraction of Treatment Threshold at Baseline and Change in Number of Clinical Risk Factors (CRFs) Stratified as Decreases (–1), No Change (0), or Increase (+1, +2 or more)

eFigure 1. Absolute and Relative Change in Major Osteoporotic Fracture (MOF) Risk and Hip Fracture Risk for Increasing Intervals Between Fracture Risk Assessments According to Change in the Number of Clinical Risk Factors (CRFs) Stratified as Decrease (–1), No Change (0), or Increase (+1, +2 or more)

eFigure 2. Importance of Variables Predicting Transition to High Fracture Risk According to Fixed 20% Major Osteoporotic Fracture (MOF) Risk, Fixed 3% Hip Fracture Risk, and Age-dependent MOF Risk

This supplementary material has been provided by the authors to give readers additional information about their work.

© 2020 Leslie WD et al. *JAMA Network Open*.

eTable 1. Number (Percent) Reaching High Fracture Risk at Follow-up According to Fraction of Treatment Threshold at Baseline and Change in Number of Clinical Risk Factors (CRFs)

Criterion for high fracture risk	Change in CRFs	<25% threshold	25-49% threshold	50-74% threshold	75-99% threshold	Combined
Fixed MOF treatment threshold	Decrease	0 / 92 (0)	S / 312 (<1.0)	10* / 189 (4.0)	14 / 81 (17.3)	22 / 674 (3.3)
(10-year fracture risk 20%) [a]	No change	0 / 1658 (0)	15* / 4034 (<1.0)	60* / 1320 (5.0)	178 / 419 (42.5)	253 / 7431 (3.4)
	Increase	S / 545 (<1.0)	70* / 1136 (6.3)	155 / 332 (46.7)	85 / 117 (72.6)	415 / 2427 (17.1)
	Overall	16 / 2388 (0.7)	142 / 5649 (2.5)	249 / 1872 (13.3)	283 / 623 (45.4)	690 / 10532 (6.6)
Fixed hip fracture treatment threshold	Decrease	S / 300 (<1.0)	15* / 146 (9.6)	21 / 81 (25.9)	21 / 43 (48.8)	58 / 570 (10.2)
(10-year fracture risk 3%) [a]	No change	40* / 3986 (1.0)	189 / 1569 (12.0)	316 / 774 (40.8)	316 / 433 (73.0)	864 / 6762 (12.8)
	Increase	90 / 1188 (7.6)	155 / 416 (37.3)	164 / 214 (76.6)	99 / 113 (87.6)	624 / 2209 (28.2)
	Overall	168 / 5649 (3.0)	412 / 2202 (18.7)	522 / 1093 (47.8)	444 / 597 (74.4)	1546 / 9541 (16.2)
Age-dependent MOF treatment threshold [a]	Decrease	0 / 9 (0)	0 / 185 (0)	7 / 217 (3.2)	16 / 143 (11.2)	23 / 554 (4.2)
	No change	0 / 29 (0)	6 / 3429 (0.2)	51 / 2715 (1.9)	204 / 880 (23.2)	261 / 7053 (3.7)
	Increase	0 / 18 (0)	59 / 1031 (5.7)	277 / 758 (36.5)	159 / 250 (63.6)	648 / 2349 (27.6)
	Overall	0 / 58 (0)	122 / 4804 (2.5)	414 / 3801 (10.9)	396 / 1293 (30.6)	932 / 9956 (9.4)

S, suppressed small numbers or * rounded due to small numbers. [a] Major osteoporotic fracture (MOF) and hip fracture risk computed using the FRAX tool with bone mineral density (BMD).

© 2020 Leslie WD et al. *JAMA Network Open*.

eTable 2. Time in Years (95% CI) for 5% of the Population to Reach High Fracture Risk According to Fraction of Treatment Threshold at Baseline and Change in Number of Clinical Risk Factors (CRFs)

	Fixed MOF threshold 20%			
Change in CRFs	<25% threshold	25-49% threshold	50-74% threshold	75-99% threshold
Decrease	>15 (>15, >15)	14.6 (12.5, >15)	6.4 (4.9, 10.4)	3.3 (2.9, 4.1)
No change	>15 (>15, >15)	14.6 (14.3, >15)	5.2 (4.7, 5.9)	2.5 (2.3, 2.8)
Increase	10.2 (8.8, 13.1)	5.2 (4.8, 5.8)	2.9 (2.8, 3.0)	2.4 (2.3, 2.8)
Overall	14.5 (13.1, >15)	8.5 (7.8, 9.1)	3.8 (3.6, 4.0)	2.7 (2.4, 2.8)
	Fixed hip fracture threshold 3%			
Change in CRFs	<25% threshold	25-49% threshold	50-74% threshold	75-99% threshold
Decrease	14.0 (10.3, >15)	4.6 (3.9, 6.4)	2.9 (2.8, 3.4)	2.7 (2.4, 3.0)
No change	11.8 (11, 13.6)	4.0 (3.8, 4.3)	2.7 (2.6, 2.8)	2.1 (1.9, 2.3)
Increase	6.0 (5.6, 6.5)	3.0 (2.9, 3.1)	2.7 (2.4, 2.8)	2.5 (2.3, 2.7)
Overall	8.5 (8, 9)	3.5 (3.4, 3.7)	2.7 (2.6, 2.8)	2.3 (2.1, 2.4)
	Age-dependent MOF threshold			
Change in CRFs	<25% threshold	25-49% threshold	50-74% threshold	75-99% threshold
Decrease	>15 (>15, >15)	>15 (>15, >15)	6.8 (5.0, 13.1)	3.7 (3.2, 5)
No change	>15 (>15, >15)	>15 (>15, >15)	8.8 (7.6, 10.8)	2.7 (2.6, 2.9)
Increase	>15 (>15, >15)	4.7 (4.3, 5.2)	2.7 (2.5, 2.8)	2.2 (2.0, 2.4)
Overall	>15 (>15, >15)	8.8 (8.1, 9.8)	3.9 (3.7, 4.1)	2.7 (2.5, 2.8)

>15 indicates that less than 5% of the population reached high fracture risk by 15 years.

© 2020 Leslie WD et al. *JAMA Network Open*.

eTable 3. Time in Years (95% CI) for 20% of the Population to Reach High Fracture Risk According to Fraction of Treatment Threshold at Baseline and Change in Number of Clinical Risk Factors (CRFs)

	Fixed MOF threshold 20%			
Change in CRFs	<25% threshold	25-49% threshold	50-74% threshold	75-99% threshold
Decrease	>15 (>15, >15)	>15 (>15, >15)	11.9 (9.2, 14.6)	5.6 (4.6, 7.4)
No change	>15 (>15, >15)	>15 (>15, >15)	9.7 (8.9, 11.4)	3.7 (3.5, 4)
Increase	14.6 (14.4, >15)	9.7 (9.1, 10.6)	4.4 (4.1, 4.8)	3.7 (3.4, 4)
Overall	>15 (>15, >15)	13.7 (13.2, 14.5)	6.7 (6.4, 7.1)	3.9 (3.7, 4.1)
	Fixed hip fracture threshold 3%			
Change in CRFs	<25% threshold	25-49% threshold	50-74% threshold	75-99% threshold
Decrease	>15 (14.9, >15)	8.7 (7.3, 11.1)	4.8 (4.1, 6)	4.0 (3.4, 4.9)
No change	>15 (14.9, >15)	7.5 (7.1, 8)	4.0 (3.8, 4.2)	3.2 (3.1, 3.3)
Increase	10.9 (10.2, 11.6)	4.9 (4.5, 5.3)	3.8 (3.6, 4.1)	3.6 (3.4, 3.9)
Overall	14.5 (13.9, 14.9)	6.4 (6.2, 6.7)	4.0 (3.8, 4.1)	3.3 (3.2, 3.5)
	Age-dependent MOF threshold			
Change in CRFs	<25% threshold	25-49% threshold	50-74% threshold	75-99% threshold
Decrease	>15 (>15, >15)	>15 (>15, >15)	14.5 (11.7, 15)	7.9 (6.2, 11.3)
No change	>15 (>15, >15)	>15 (>15, >15)	>15 (14.9, >15)	4.7 (4.4, 5.0)
Increase	>15 (>15, >15)	10.8 (9.6, 12)	4.6 (4.3, 4.9)	3.5 (3.3, 3.8)
Overall	>15 (>15, >15)	>15 (14.9, >15)	8.0 (7.5, 8.5)	4.6 (4.3, 4.8)

>15 indicates that less than 20% of the population reached high fracture risk by 15 years.

© 2020 Leslie WD et al. *JAMA Network Open*.

eTable 4. Time in Years (95% CI) for 50% of the Population to Reach High Fracture Risk According to Fraction of Treatment Threshold at Baseline and Change in Number of Clinical Risk Factors (CRFs)

	Fixed MOF threshold 20%			
Change in CRFs	<25% threshold	25-49% threshold	50-74% threshold	75-99% threshold
Decrease	>15 (>15, >15)	>15 (>15, >15)	14.6 (13.5, >15)	9.2 (7.4, 11.6)
No change	>15 (>15, >15)	>15 (>15, >15)	14.5 (13.3, 14.6)	5.9 (5.4, 6.2)
Increase	>15 (>15, >15)	14.5 (13.5, 14.6)	7.4 (6.8, 7.7)	5.9 (5.2, 6.4)
Overall	>15 (>15, >15)	>15 (>15, >15)	10.9 (9.9, 11.5)	6.2 (5.8, 6.5)
	Fixed hip fracture threshold 3%			
Change in CRFs	<25% threshold	25-49% threshold	50-74% threshold	75-99% threshold
Decrease	>15 (>15, >15)	13.5 (11.2, 14.9)	8 (6.6, 9.2)	6.6 (5.5, 7.7)
No change	>15 (>15, >15)	11.8 (11.1, 12.3)	6.7 (6.2, 6.8)	5 (4.5, 5.2)
Increase	14.9 (14.7, 14.9)	8.0 (7.4, 8.3)	6.4 (5.8, 6.6)	6 (5.4, 6.4)
Overall	>15 (>15, >15)	10.3 (9.7, 10.5)	6.6 (6.2, 6.7)	5.4 (5.0, 5.5)
	Age-dependent MOF threshold			
Change in CRFs	<25% threshold	25-49% threshold	50-74% threshold	75-99% threshold
Decrease	>15 (>15, >15)	>15 (>15, >15)	>15 (>15, >15)	14.5 (12, >15)
No change	>15 (>15, >15)	>15 (>15, >15)	>15 (>15, >15)	9 (8.1, 9.5)
Increase	>15 (>15, >15)	>15 (14.9, >15)	8.7 (8.0, 9.0)	6.1 (5.5, 6.5)
Overall	>15 (>15, >15)	>15 (>15, >15)	14.1 (13, 14.5)	8.3 (7.5, 8.5)

>15 indicates that less than 50% of the population reached high fracture risk by 15 years.

© 2020 Leslie WD et al. *JAMA Network Open*.

eTable 5. Number (Percent) Reaching High Fracture Risk at Follow-up According to Fraction of Treatment Threshold at Baseline and Change in Number of Clinical Risk Factors (CRFs) Stratified as Decreases (–1), No change (0), or Increase (+1, +2, or more)

Criterion for high fracture risk	Change in CRFs	<25% threshold	25-49% threshold	50-74% threshold	75-99% threshold	Combined
Fixed MOF treatment threshold 20% [a]	-1	0 / 92 (0)	S / 312 (<1)	~10 / 189 (4)	14 / 81 (17.3)	22 / 674 (3.3)
	0	0 / 1658 (0)	~15 / 4034 (<1)	~60 / 1320 (5)	178 / 419 (42.5)	253 / 7431 (3.4)
	+1	S / 545 (<1)	~70 / 1136 (6.3)	155 / 332 (46.7)	85 / 117 (72.6)	313 / 2130 (14.7)
	+2 or more	~15 / 93 (15)	57 / 167 (34.1)	25 / 31 (80.6)	6 / 6 (100.0)	102 / 297 (34.3)
	Overall	16 / 2388 (0.7)	142 / 5649 (2.5)	249 / 1872 (13.3)	283 / 623 (45.4)	690 / 10532 (6.6)
Fixed hip fracture treatment threshold 3% [a]	-1	S / 300 (<1)	~15 / 146 (9.6)	21 / 81 (25.9)	21 / 43 (48.8)	58 / 570 (10.2)
	0	~40 / 3986 (1)	189 / 1569 (12.0)	316 / 774 (40.8)	316 / 433 (73.0)	864 / 6762 (12.8)
	+1	90 / 1188 (7.6)	155 / 416 (37.3)	164 / 214 (76.6)	99 / 113 (87.6)	508 / 1931 (26.3)
	+2 or more	33 / 175 (18.9)	54 / 71 (76.1)	21 / 24 (87.5)	8 / 8 (100)	116 / 278 (41.7)
	Overall	168 / 5649 (3.0)	412 / 2202 (18.7)	522 / 1093 (47.8)	444 / 597 (74.4)	1546 / 9541 (16.2)
Age-dependent MOF treatment threshold [a]	-1	0 / 9 (0)	0 / 185 (0)	7 / 217 (3.2)	16 / 143 (11.2)	23 / 554 (4.2)
	0	0 / 29 (0)	6 / 3429 (0.2)	51 / 2715 (1.9)	204 / 880 (23.2)	261 / 7053 (3.7)
	+1	0 / 18 (0)	59 / 1031 (5.7)	277 / 758 (36.5)	159 / 250 (63.6)	495 / 2057 (24.1)
	+2 or more	0 / 2 (0)	57 / 159 (35.8)	79 / 111 (71.2)	17 / 20 (85.0)	153 / 292 (52.4)
	Overall	0 / 58 (0)	122 / 4804 (2.5)	414 / 3801 (10.9)	396 / 1293 (30.6)	932 / 9956 (9.4)

S, suppressed small numbers and ~, rounded due to small numbers. [a] Major osteoporotic fracture (MOF) and hip fracture risk computed using the FRAX tool with bone mineral density (BMD).

© 2020 Leslie WD et al. *JAMA Network Open*.

eFigure 1. Absolute and Relative Change in Major Osteoporotic Fracture (MOF) Risk and Hip Fracture Risk for Increasing Intervals Between Fracture Risk Assessments According to Change in the Number of Clinical Risk Factors (CRFs) Stratified as Decrease (–1), No Change (0), or Increase (+1, +2 or more).

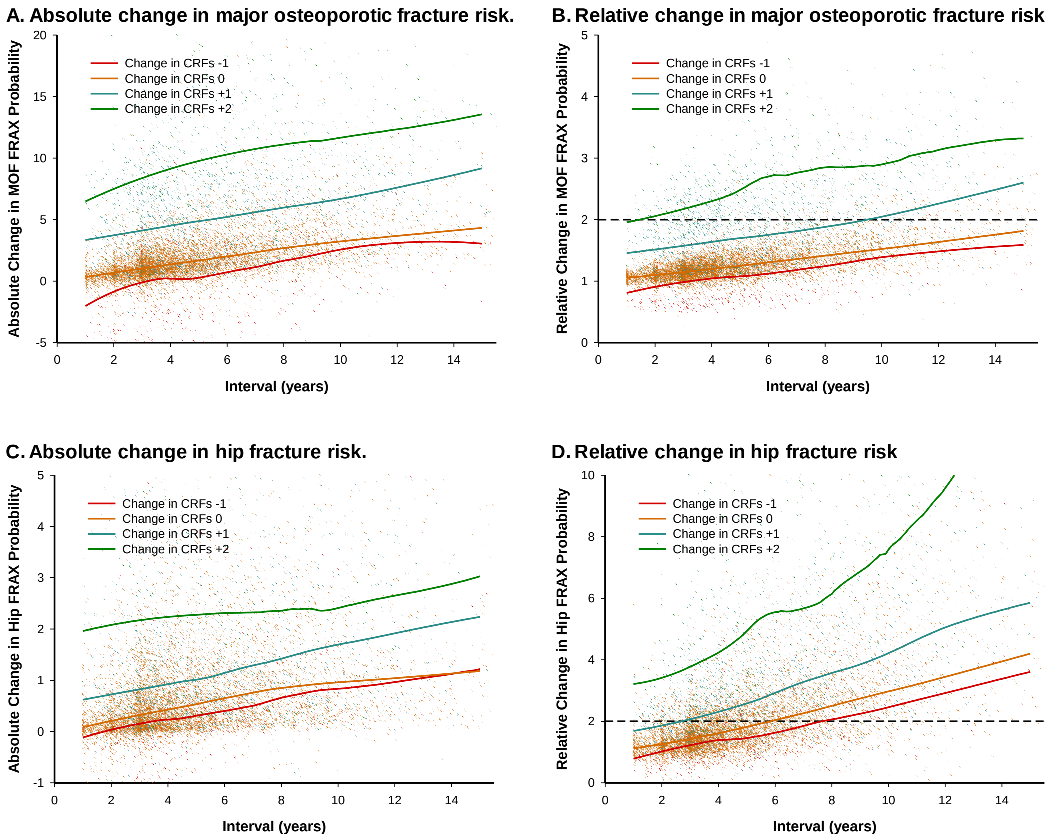

Solid lines are loess smoothed curves fitted to the dots which are individual patient observations. Dashed line (right hand) indicates a doubling in baseline fracture risk.

© 2020 Leslie WD et al. *JAMA Network Open*.

eFigure 2. Importance of Variables Predicting Transition to High Fracture Risk According to Fixed 20% Major Osteoporotic Fracture (MOF) Risk, Fixed 3% Hip Fracture Risk, and Age-dependent MOF Risk

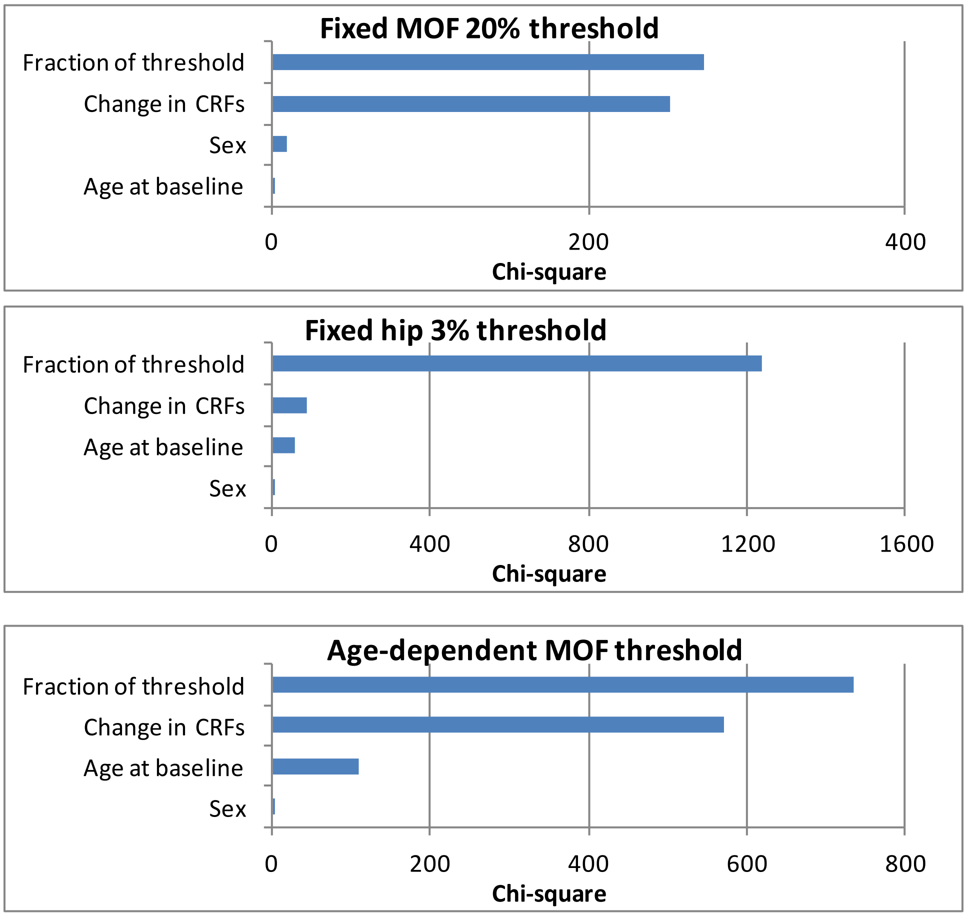

Data from Cox regression models. Rank ordered, highest to lowest model Chi-square statistic.

© 2020 Leslie WD et al. *JAMA Network Open*.
